# TFAM in mtDNA Homeostasis: Open Questions

**DOI:** 10.3390/dna3030011

**Published:** 2023-08-07

**Authors:** Mikhail Alexeyev

**Affiliations:** Department of Physiology and Cell Biology, University of South Alabama, Mobile, AL 36688, USA

Transcription factor A, mitochondrial (TFAM) is a key player in mitochondrial DNA (mtDNA) transcription and replication. It was first isolated in 1988 as an activator of mitochondrial transcription [1] and was later shown to play multiple other roles, including critical functions in mtDNA replication and compaction into nucleoids. Since then, our understanding of this protein’s contributions to mtDNA homeostasis has greatly increased. However, many questions remain to be answered. Here, I highlight four categories of outstanding questions.

1. No dedicated DNA primase has been identified in mitochondria to date. Therefore, mitochondrial transcription is believed to be responsible for the synthesis of primers for mtDNA replication. This concept mechanistically links mtDNA transcription and replication. Indeed, cells with the inactivation of key components of the mtDNA transcription apparatus (TFAM, POLRMT, or TFB2M) remain viable but show a loss of mtDNA [2,3]. In the best-understood, most widely accepted strand-asynchronous mtDNA replication model, the leading mtDNA strand replication is primed using a mitochondrial light strand promoter (LSP) transcript [4–6]. In vitro, LSP transcription appears to depend on TFAM, particularly on its C-terminus [7]. However, more recent evidence has suggested that tail-less TFAM can initiate LSP transcription on longer DNA templates [8,9] and can support mtDNA transcription and replication in situ [10]. Notably, the initiation of the lagging mtDNA strand replication appears to be TFAM-independent [11,12]. Whether TFAM is required for priming mtDNA replication in alternative modes remains unclear [13,14]. However, the mtDNA loss observed after TFAM inactivation suggests that (1) in these modes, mtDNA replication is TFAM-dependent; (2) these modes are accessory and could not substitute for strand-asynchronous replication; and (3) in TFAM knockouts, mtDNA loss results from the loss of TFAM-dependent nucleoid organization leading to mtDNA degradation, rather than from an inability to initiate replication. These possibilities await further investigation.
2. In many studies, similarly sized nucleoid-like structures containing DNA but little to no TFAM, or vice versa, have been observed within the same cell [15–19]. According to the current model, the TFAM content of nucleoids and the extent of mtDNA compaction depend on TFAM abundance. If so, what is the mechanistic basis underlying the variability in TFAM abundance in these structures within one cell, where TFAM is presumably uniformly distributed? Are the processes of nucleoid TFAM entry and exit regulated? If so, how?
3. The availability of crystal structures of TFAM in complex with LSP, mitochondrial heavy strand promoter 1 (HSP1), nonspecific DNA, and transcription initiation complexes (ICs) at LSP and HSP1 [20–23] have greatly expanded the understanding of mitochondrial transcription. However, they have also prompted several pertinent questions requiring further investigation. Biochemical [7,23,24] and structural [20,21,23] studies have established that, in simple systems consisting of only TFAM and mitochondrial promoters, TFAM binds LSP and HSP1 in opposite orientations. Nonetheless, IC structure comprising premelted promoter DNA template, TFAM, POLRMT, and TFB2M have shown TFAM bound in the same orientation to both LSP and HSP1. In the current model of mitochondrial transcription, transcription complexes assemble at mitochondrial promoters sequentially. First, TFAM binds upstream of a promoter, and POLRMT and TFB2M are subsequently recruited, presumably, through interaction with TFAM’s C-terminal tail [25–27]. However, recent in situ data have indicated that TFAM’s C-terminal tail is dispensable for mtDNA transcription and replication [10]. In addition, tail-less TFAM has been found to support transcription at LSP in vitro when a larger template is used [8,9]. Therefore, how tail-less TFAM recruits POLTMT and TFB2M to mitochondrial promoters remains unclear. Further clarification is also required in terms of when and how TFAM reverses its orientation on HSP1 during IC assembly. Does this reversal occur before or after the recruitment of POLRMT/TFB2M? If before, what causes TFAM to reattach in the opposite orientation at the same site? If after, what happens to POLRMT/TFB2M after TFAM detaches? It is conceivable that the orientation of TFAM binding at HSP1 is modulated by one or several accessory factors (e.g., POLRMT and/or TFB2M), which direct TFAM to bind in the opposite orientation after the factor-less TFAM dissociates from the promoter through normal on–off processes. However, this hypothesis requires experimental validation.
4. What is the molecular basis underlying the differential sensitivity of mitochondrial promoters to TFAM alterations, such as changes in TFAM abundance or its primary amino acid sequence? Early biochemical and structural studies have provided a coherent picture that attributed the higher sensitivity of LSP to TFAM C-terminal truncation and DNA bending defects to this promoter’s dependence on DNA bending for the proper alignment of the TFAM’s C-terminus with the transcription start site [20,21,23]. However, recent studies that demonstrated the dispensability of TFAM’s C-terminal tail and the same orientation o TFAM at LSP and HSP1 put this rationale into question.

Answers to these and other pertinent questions should greatly improve the granularity of the understanding of mtDNA homeostasis.
